# High resolution surface plasmon resonance imaging for single cells

**DOI:** 10.1186/1471-2121-15-35

**Published:** 2014-12-01

**Authors:** Alexander W Peterson, Michael Halter, Alessandro Tona, Anne L Plant

**Affiliations:** Biosystems and Biomaterials Division, National Institute of Standards and Technology, 100 Bureau Drive, Mail Stop 8313, Gaithersburg, MD 20899 USA

**Keywords:** Surface plasmon, Imaging, Microscope, Resolution, Cells, Focal adhesions, Penetration depth, Microspheres

## Abstract

**Background:**

Surface plasmon resonance imaging (SPRI) is a label-free technique that can image refractive index changes at an interface. We have previously used SPRI to study the dynamics of cell-substratum interactions. However, characterization of spatial resolution in 3 dimensions is necessary to quantitatively interpret SPR images. Spatial resolution is complicated by the asymmetric propagation length of surface plasmons in the x and y dimensions leading to image degradation in one direction. Inferring the distance of intracellular organelles and other subcellular features from the interface by SPRI is complicated by uncertainties regarding the detection of the evanescent wave decay into cells. This study provides an experimental basis for characterizing the resolution of an SPR imaging system in the lateral and distal dimensions and demonstrates a novel approach for resolving sub-micrometer cellular structures by SPRI. The SPRI resolution here is distinct in its ability to visualize subcellular structures that are in proximity to a surface, which is comparable with that of total internal reflection fluorescence (TIRF) microscopy but has the advantage of no fluorescent labels.

**Results:**

An SPR imaging system was designed that uses a high numerical aperture objective lens to image cells and a digital light projector to pattern the angle of the incident excitation on the sample. Cellular components such as focal adhesions, nucleus, and cellular secretions are visualized. The point spread function of polymeric nanoparticle beads indicates near-diffraction limited spatial resolution. To characterize the z-axis response, we used micrometer scale polymeric beads with a refractive index similar to cells as reference materials to determine the detection limit of the SPR field as a function of distance from the substrate. Multi-wavelength measurements of these microspheres show that it is possible to tailor the effective depth of penetration of the evanescent wave into the cellular environment.

**Conclusion:**

We describe how the use of patterned incident light provides SPRI at high spatial resolution, and we characterize a finite limit of detection for penetration depth. We demonstrate the application of a novel technique that allows unprecedented subcellular detail for SPRI, and enables a quantitative interpretation of SPRI for subcellular imaging.

**Electronic supplementary material:**

The online version of this article (doi:10.1186/1471-2121-15-35) contains supplementary material, which is available to authorized users.

## Background

A critical measurement challenge in cell biology is to quantify the interaction of cells with their extracellular matrix (ECM). Cells interact with and respond to their extracellular environment through surface receptors, and specifically interact with insoluble ECM proteins by ligation through integrin receptors [1, 2]. As a result of integrin binding events, intracellular proteins assemble into focal adhesions which influence cytoskeleton organization and can affect intracellular signaling pathways that control gene expression and complex responses such as proliferation and differentiation [3–6]. In addition, cells actively secrete, remodel, and modify their extracellular environment to affect physiologic processes including wound healing, development, and tumor metastasis [7–9].

Surface plasmon resonance (SPR) is generated at a thin metal surface by the coupling of incident light into the surface plasmons of the metal at an appropriate incident angle. A minimum in the reflectance of the incident light from this surface occurs at the angle where maximum plasmon coupling occurs. This angle of incidence at which there is maximum coupling into the surface plasmons is highly sensitive to the refractive index of the dielectric material at the interface. The resulting evanescent wave decays exponentially in the direction perpendicular to the sample interface and can vary in decay length from tens to hundreds of nanometers depending on the optical properties of the metal and the dielectric, and on the incident wavelength of light [10]. SPR is often used as a highly sensitive way to quantify proteins and other biological molecules at surfaces. When used in an imaging mode, SPR is an attractive technique for quantifying and visualizing cells and their extracellular environment because it is label-free, requires low levels of incident light to achieve good contrast, and is able to quantitatively report on very small amounts (3 ng/cm^2^) of biological material [11, 12]. Our work in this field has advanced the spatial resolution of SPRI to unprecedented levels.

While SPRI can provide useful information on fixed cells, previous work in our laboratory has also demonstrated the imaging of live cells with SPRI for visualizing cell-substrate adhesion dynamics, and the dynamics of deposition of cellular ECM proteins during matrix remodeling [11, 13]. The work was performed with a prism configuration which provided relatively low effective magnification, comparable to a microscope with 5× magnification and ≈ 3 μm spatial resolution. Here, we demonstrate achieving near diffraction limited resolution by employing SPRI through a high numerical aperture objective lens. The current system is constructed on an inverted microscope platform, so it has the additional advantage of allowing other imaging modalities. In the current configuration, we use a digital light projector to pattern the incident light on the back focal plane (BFP) of the objective. The spatial filtering of light allows control over the angle with which the incident light hits the surface, to maximize the efficiency of coupling with plasmons to produce SPR. Using this optical setup, we are able to visualize unprecedented intracellular detail. Putative cell-matrix adhesions are observed as areas of higher signal intensity than neighboring areas, as would be expected from dense proteinaceous regions. The resolution appears comparable to other surface sensitive techniques such as TIRF microscopy, but the technique has the advantage of imaging without the need for any added labeling. We also resolve intracellular organelles such as the cell nucleus, which indicates that the nucleus resides within the evanescent field.

Here we ask: how far can we detect the evanescent field propagation into the cell, and how close are these intracellular features to the substratum from which the evanescent wave originates? We demonstrate a method for using polymer microspheres to effectively measure the evanescent wave penetration depth in the imaging mode. We show, through measurement of these reference beads, that the theoretically calculated field decay length, described by 1/e, serves as an accurate imaging threshold for the detection limit of the SPR penetration depth.

## Results

### SPR imaging system design

**Figure 1:**
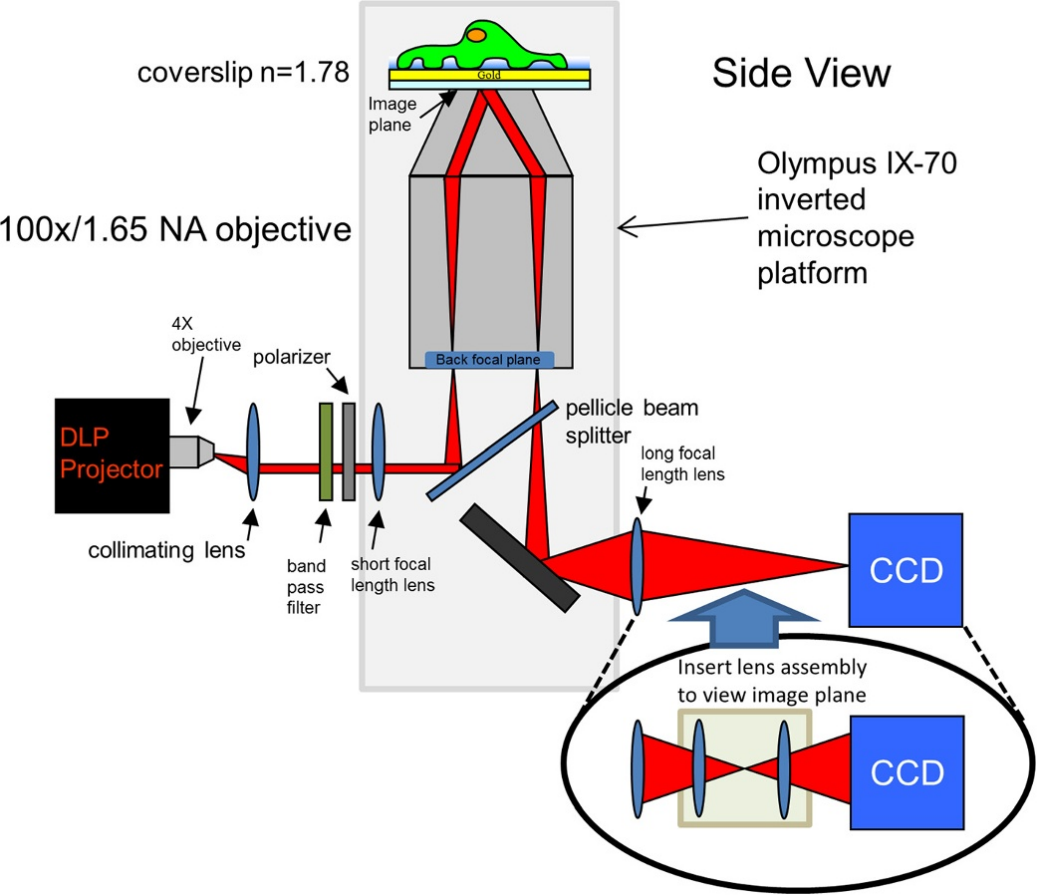
**SPR imaging through a microscope objective instrumentation schematic.** Incident angle selected light is generated and patterned by a digital light projector that is then collimated, wavelength filtered, linear polarized, and then directed through an inverted microscope platform (shaded in grey). The reflected image is captured on a CCD camera after a switchable lens assembly selects for either the image plane or back focal plane.

The use of an objective lens for exciting SPR is described schematically in Figure 1. The microscope objective and inverted microscope body were designed for total internal reflection fluorescence microscopy (TIRFM) [14] of cellular features. Here we adapted this configuration for SPRI. For performing SPRI, the microscope objective is used to direct and collect the incident and reflected light. The use of the objective lens and microscope body allows for SPR to be integrated into a device that allows multiple imaging modes.

Here we use a digital light projector (DLP) to provide an incoherent white light source, a filter wheel for specific wavelength selection, and a switchable lens system that allows projection of either the sample image plane or the back focal plane onto the CCD camera. The DLP allows for computer controlled spatial selection and positioning of the incident light for precise control of the incident angle for maximum SPR coupling. The image projected by the DLP is de-magnified by 1/3 using the positioning of two lenses as described in the Methods section and Additional file 1, resulting in projection of the resized image from the DLP onto the back aperture of the microscope objective (i.e., the back focal plane). Having precise control of incident light with the digital light source, the ability to select specific wavelengths of light, and the ability to capture both the sample image and back focal plane image, allows for the systematic evaluation of how SPR image contrast and resolution at the specimen plane is effected by the spectral properties and the incident angle of the illumination light.

### Back focal plane imaging and excitation angle selection

**Figure 2:**
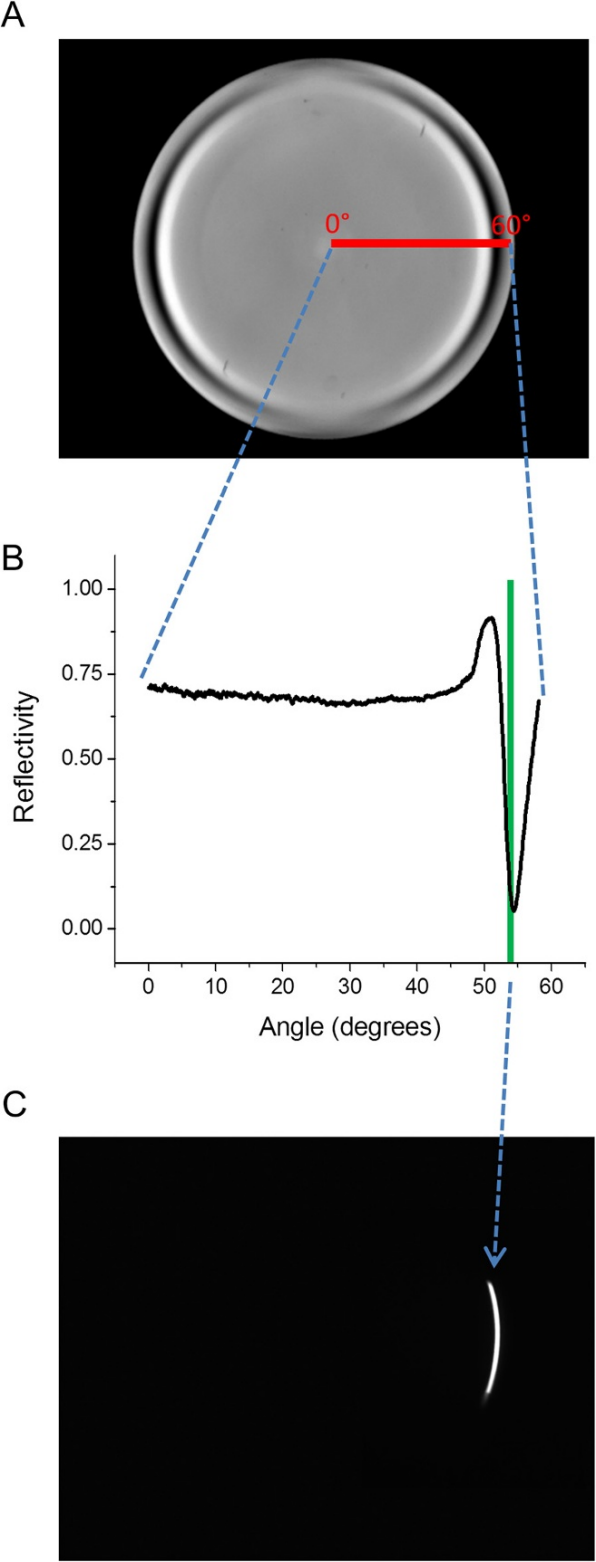
**Back focal plane (BFP) image of SPR angle reflectance data and demonstration of incident angle control of excitation light by digital light projector (DLP). A)** The BFP is fully illuminated, at all incident angles available, by 620 nm excitation light off of a 45 nm gold coated coverslip under water media. The incident light is linearly polarized in the x-direction. The dark crescent shaped regions at the edges of the field are where the incident light passes through the objective lens at a high angle of incidence and is coupled into the surface plasmons of the metal film, thus reducing reflectance. The absorbance occurs at the sides of the field where the incident light is p-polarized, and not at the top and bottom of the field where the incident light is s-polarized. The red line traces the angular distribution of incident light onto the image plane from the center of the BFP (0°) to the periphery (60°). **B)** A line scan of the red line in **A)** in the p-polarized direction shows the angular dependence of reflectivity and SPR coupling. The green line depicts the angle of illumination that provides maximum SPR coupling (≈53.5° for 620 nm) to be used for SPR imaging. **C)** Same BFP image area as shown in **A)** except here the DLP is used to project a thin arc of light that contains the same radial incident angle (≈53.5° illumination angle shown) as depicted by the green line in **B)**.

The spatial location of light at the back focal plane (BFP) of the microscope objective indicates the incident angle with which that light impinges on the sample. The angle of incidence increases approximately linearly with distance from the optical axis of the objective. For example, an illuminated spot in the center of the BFP would irradiate the sample normal to the surface while a spot at the edge of the objective BFP would be illuminating the sample at an angle that is steep enough to achieve total internal reflection. This phenomenon is shown in Figure 2A, which displays the image of the BFP which is fully illuminated by the reflection of 620 nm from a 45 nm thick gold coating on a glass coverslip in buffer. The incident light is linearly polarized, and impinges on the sample at angles that vary from 0° at the center of the BFP to an angle of 60° at the periphery. This angle span is marked by the red line (Figure 2A). The regions of lowest intensity, which appear as the dark ring at the periphery of the BFP, occur at the angle of incidence where the light is maximally coupled with the surface plasmons, and minimum reflectance occurs. This angle is ≈ 54.0°. The strong plasmon absorbance of the p-polarized light occurs in the x-direction and fades away azimuthally as the light becomes entirely s-polarized in the y-direction.

A line scan from the center to the periphery of the BFP captures the angular dependence of the surface plasmon resonance and this dependence is shown in Figure 2B. This angle scan can be fit by, and has good agreement with, a 3-layer Fresnel model assuming published values for the prism/gold/water interface (Additional file 2) [15–18]. The green line represents the angle (≈53.5°) of excitation used for SPR imaging at 620 nm.

Figure 2C shows the shape of the reflected light from the BFP when patterned illumination is provided by the DLP to achieve maximum surface plasmon excitation. The shape of the excitation light projected onto the BFP for SPR coupling at ≈ 53.5° is a crescent shape arc of light that spans ≈ 40° azimuthal (essentially identical to the reflected image shown). Although not demonstrated rigorously here, the selection of the characteristics of this crescent shaped pattern of incident light for SPR imaging was the result of an optimization study for image quality and contrast (Additional file 3). Qualitatively, we observed that increasing the radial width of the crescent beyond a certain amount decreased the image contrast. The azimuthal length of the crescent arc also appeared to control image quality: lengthening the crescent resulted in lower background noise, up to limit, ≈40°, whereupon increased length then decreased image contrast. Both of these effects are likely caused by an increase in background signal by rays that do not contribute to the useful SPR signal at a specific incident angle. Shortening the crescent shape decreased image quality and eventually resulted in the appearance of rings, interference patterns, and background unevenness characteristic of coherent laser light illumination sources.

### SPR imaging of cells

**Figure 3:**
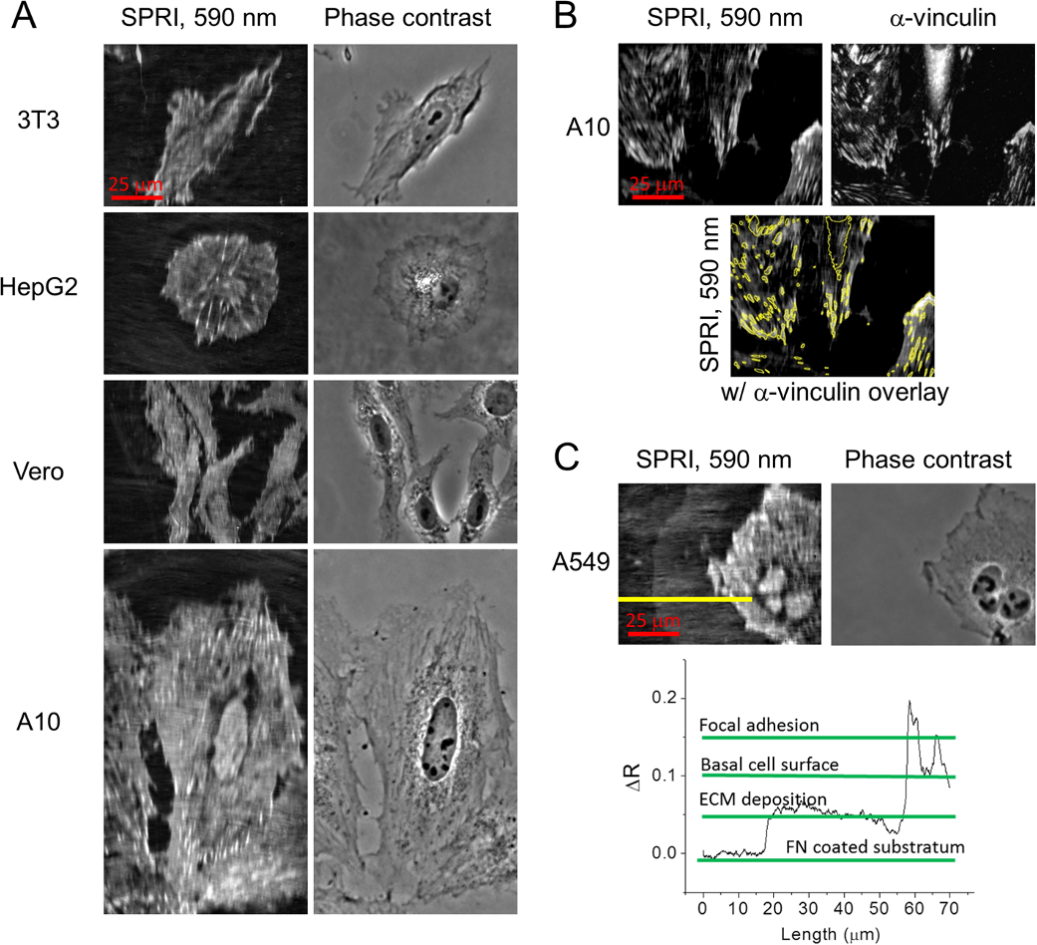
**SPR and phase contrast images of five cell types (3T3, HepG2, Vero, A10 and A549) fixed under PBS buffer 72 h after plating on fibronectin coated substrates, and SPR image comparison with fluorescently stained α-vinculin. A)** SPR images collected with 100X/1.65 NA objective using 590 nm incident light; phase contrast images acquired with a 20X/0.4 NA objective. The SPR image displays distinct changes in reflectivity for various intracellular components within the evanescent wave such as cell membrane, focal adhesions, and cell nucleus. **B)** SPR image of A10 cell collected as in **A)** but contrast adjusted on a linear scale to emphasize the focal adhesions. Subsequently, the sample was immunofluorescently labeled with α-vinculin and imaged with a 63X/1.3 NA objective. A manual intensity threshold of α-vinculin was then overlaid onto the SPR image for comparison. **C)** Imaging conditions same as in **A)**. A region of intermediate intensity, putative extracellular deposited material, is detected along the cell periphery. A linescan in the SPR image is segregated into four distinct image features: fibronectin (FN) coated substratum, extracellular deposited material (ECM), the basal cell surface, and focal adhesions. The scale bar of 25 μm applies to all images in figure.

Figure 3A and 3B shows SPR and phase contrast images of five selected cell types: mouse fibroblast cells (3T3), human liver carcinoma cells (HepG2), kidney epithelial cells (Vero), vascular smooth muscle cells (A10) and adenocarcinomic alveolar basal epithelial cells (A549) that were seeded and fixed after 72 h on a fibronectin coated substrate as described in Methods. The left row shows SPR images taken with 590 nm incident light in comparison to the right row using phase contrast. The SPR images show high optical contrast of the cell-substrate interface with a spatial resolution sufficient to reveal a number of subcellular features. The brighter regions of the interest are areas where the density of dielectric material is greater, resulting in greater reflectivity of the incident light due to reduced plasmon coupling. Thus the optical contrast is measured in reflectivity units that have a direct relationship to the mass and refractive index of material within the evanescent wave. Since values used for refractive index for proteins and lipids are usually similar [19], it is likely that the difference in mass, not refractive index, is the primary source of contrast. However, because the thickness of the cell is much larger than the depth of penetration of the evanescent wave, the reflectivity contrast will also be determined by the distance between the cell components and the surface.

The optical contrast in the SPR images is sufficient to define the edge of the cell with relatively high signal-to-noise and this facilitates image segmentation of the cell object [11]. SPR images show punctate regions of high reflectivity that are putatively the cellular focal adhesions. Focal adhesions are known to be regions of high protein density [20] and are expected to reside near the cell-substratum interface. Cytoskeletal structure appears to be visible especially in the A10 image shown. There are also lower intensity regions visualized within the cell that indicate regions of either lower protein density or greater distance from the substrate.

It is notable that different cell types present distinctly different features when observed with SPRI. For some of the cell types shown here (Vero, A10, A549), the SPR images allow visualization of the nucleus of the cell. The ability to visualize the nucleus indicates that it is sufficiently close to the cell-substratum interface such that it is in the evanescent field; and that the nucleus contains sufficient density of proteins and nucleic acids as to have a refractive index that is distinct enough from the cytoplasm to be distinguishable. Also, as best exemplified in the A549 SPR image, there appears to be a region of low contrast surrounding the nucleus. This could be due to a lower refractive index due to a lower protein density in the cytoplasm immediately surrounding the nucleus.

Figure 3B, shows a comparison between an A10 cell imaged with SPR and then subsequently labeled with a fluorescent antibody for the focal adhesion associated protein vinculin, and observed by widefield fluorescence microscopy. The third image in the set depicts the outline of a threshold performed on the fluorescence image of α-vinculin overlaid on the SPR image. From this image comparison we can observe that the bright punctate regions in the SPR image match well with the α- vinculin stained regions and therefore SPRI is likely detecting focal adhesions as regions of high protein density. While significant cellular background signal is present in the fluorescence image, the SPR image shows a strong signal from the focal adhesions compared with other cellular components, such as the cell membrane.

In Figure 3C, the A549 SPR image reveals a distinct halo of signal intensity that extends to a ≈ 40 μm ring around the cell periphery. This is likely to be extracellular material that is deposited onto the fibronectin substratum by the cell. This observation is consistent with previous work in our group that measured extracellular deposition of material at the cell-periphery using a lower-resolution prism configuration SPR imaging apparatus [13]. A linescan in the SPR image is displayed in ΔR (reflectivity) units in Figure 3B, and presents four distinct image intensity thresholds that depict both extracellular and intracellular features: fibronectin coated substratum, extracellular deposited material, the basal cell surface, and protein-dense focal adhesions.

While these high resolution SPR cell images are provocative with respect to the information they apparently show, the interpretation of the images requires care. Knowledge of the lateral resolution, given by the point spread function (PSF), is needed to understand the limits of resolution. Interpretation of SPR images is also complicated by the confounding factors of refractive index, mass, and the depth of penetration for the evanescent wave. An established theoretical model to calculate the evanescent wave penetration depth has been frequently used in the past [21], and has been sufficient for most applications for SPR, such as the measurement of bio-molecular interactions that occur at surfaces well with the evanescent field. Because of the large dimensions of cells relative to the predictions of this theory, and the nature of the intracellular features that SPRI detects, it was important to establish experimentally the validity of the assumptions that are usually made about surface plasmon penetration depth. The interpretation of image features is complicated by the fact that the decay of the evanescent wave intensity with distance from the surface is exponential, and the limit of detection must consider the changes in refractive index and mass of features within the decaying evanescent field. To provide context for interpreting the subcellular features shown in Figure 3, we measured both the lateral resolution and the effective limit of detection for the evanescent field penetration depth on our high resolution SPR imaging system using polymeric reference beads.

### Lateral resolution of the high resolution SPR imaging system

**Figure 4:**
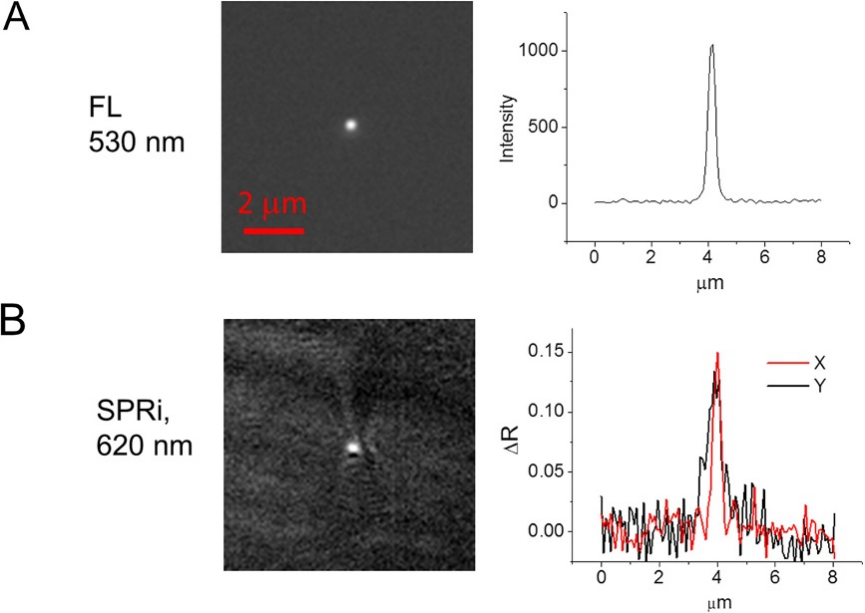
**Spatial resolution of fluorescent (FL) and SPR imaging using fluorescent point-source nanospheres. A)** Transparent coverslip a 0.17 μm diameter particle fluorescing at 515 nm peak emitting wavelength in epi-fluorescent mode. Line scan plot next to image is used to determine full width half-maximum (FWHM) at 0.29 μm for a 1.65 NA objective. **B)** SPR image of nanoparticle at 620 nm shows a FWHM of 0.3 μm in the x-direction (red) and 0.6 μm in the y-direction using the 1.65 NA objective. The scale bar of 2 μm applies to A and B. Nanoparticle measurements made under water media, and fluorescence emission collected with 530 nm bandpass filter.

The theoretical diffraction-limited resolution for a 1.65 NA microscope objective is 0.23 μm and 0.20 μm for 620 nm and 530 nm light, respectively. The theoretical propagation length for 620 nm light of the surface plasmon in the direction parallel to the surface plasmon excitation is ≈ 3 μm [21]. We measured the spatial resolution of our microscope using fluorescent nanoparticles to determine the point spread function in both epifluorescence and SPR imaging mode. We chose the fluorescence wavelength to be distinct in both excitation and emission wavelengths from the SPR imaging wavelength, and we chose the SPR wavelength for its observable lateral decay length. The fluorescent nanoparticles were measured at 530 nm emission under water in epi-fluorescent mode (Figure 4A), and a line profile plot was used to determine a full width at half maximum (FWHM) value of 0.29 μm, very close to the theoretical limit. For the second measurement, the SPR excitation light was set to 620 nm with ≈ 53.5° incident angle and the nanoparticle bead image was obtained, Figure 4B, with the corresponding line profile resulting in a FWHM of 0.30 μm in the x-direction perpendicular to the surface plasmon propagation vector, and 0.60 μm in the y-direction, parallel to the surface plasmon propagation. The asymmetry in the x- versus y- resolution arises from the surface plasmon leakage radiation decay in the direction parallel to the excitation light [22].

### Determining the effective penetration depth of the SPR evanescent field

**Figure 5:**
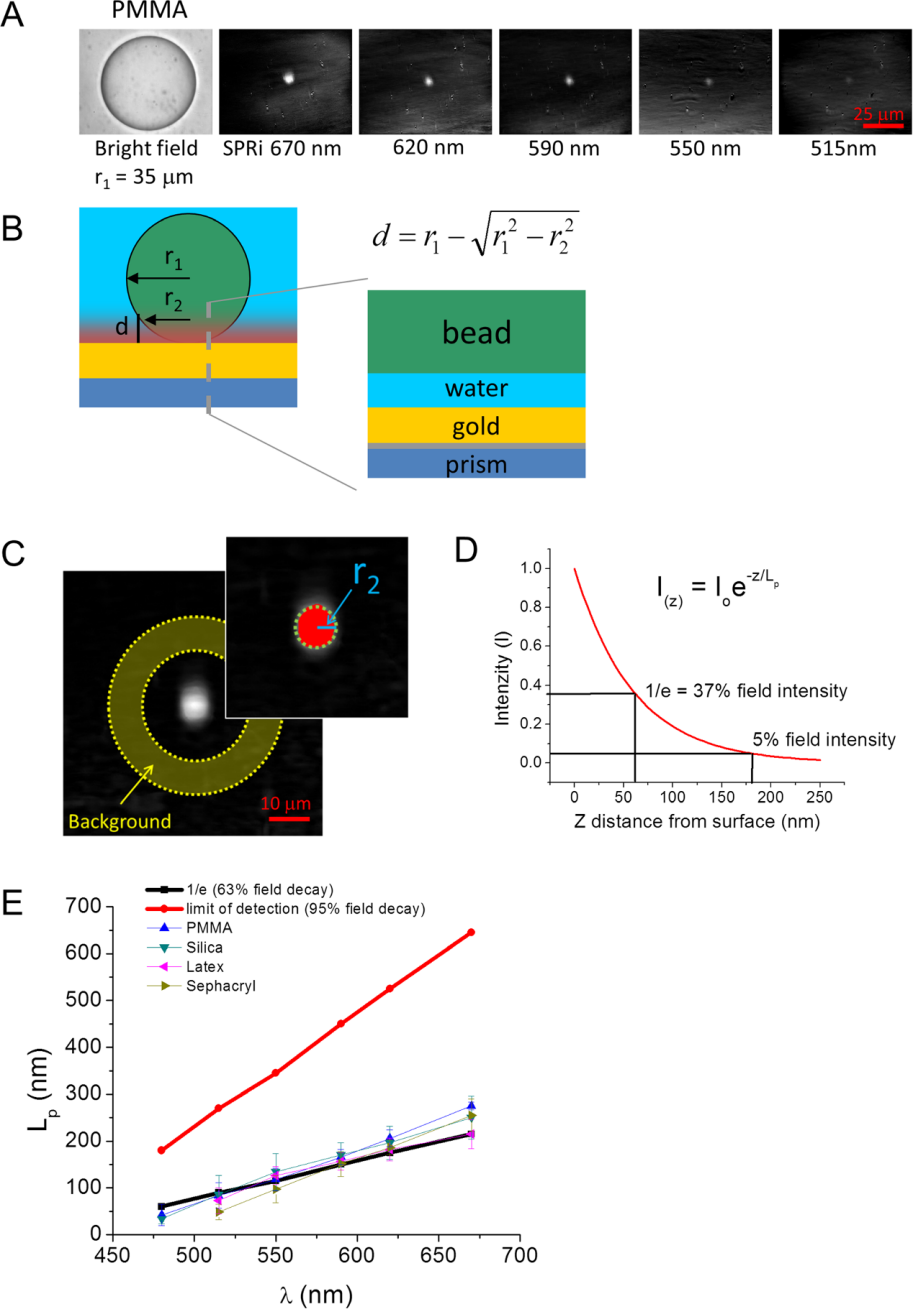
**Measurement of SPR evanescent wave (EW) penetration depth by geometric relationship of measured radii of polymer microspheres. A)** Bright-field and SPR images (for 5 wavelengths) of a polymethylmethacrylate (PMMA) microsphere in water. **B)** Diagram of a microsphere at the SPR sensor interface showing that only a fraction of the bead lies within the EW. The equation shows the relationship between the EW penetration depth (*d*) and the radius of the sphere measured by bright field (*r*_*1*_) and SPRI (*r*_*2*_). The overlay shows a layer model of the interface; the water layer decreases thickness as the bead moves toward the surface and into the EW. **C)** SPR image and insert of a microsphere used to illustrate the image analysis procedure for measuring the value of *r*_*2*_, the SPRI detected radius. The standard deviation (σ) of background intensity is determined by the annulus-shaped ROI in the primary image, and in the image insert a value of 3σ is set as the threshold with pixels values above threshold colored red; the radius of the bead (*r*_*2*_ in blue) is determined from the area of circle (green dashed circle) computed from the threshold. **D)** Exponential decay from surface into media of the SPR generated EW calculated as field intensity versus distance from surface (nm). Two values are labeled: the 1/e decay at 37% field strength commonly referred to as the “penetration depth”, and the 5% field strength value, which is the theoretical detection limit. **E)** Extent of the EW field depth (L_p_) measured for several polymer microspheres as a function of excitation wavelength, along with theoretical values of the penetration depth (1/e) and detection limit (5% field strength). Within the standard deviation of the measurement error, the decay values agree for all microspheres and the calculated penetration depth at 1/e field decay.

Theory predicts that the wavelength of incident light used to excite surface plasmons determines the evanescent wave penetration depth [21]. To directly measure the sensitivity of the SPR field as a function of distance from the surface, we employed measurements of polymer microspheres with known refractive index and diameter, and incident light of several wavelengths. Images of a representative poly (methyl methacrylate) (PMMA) microsphere are taken in bright field as well as with SPR imaging at several wavelengths (Figure 5A). The radius of the microsphere *r*_*1*_, measured in the bright field image, can be related to the radius observed in the SPR image, r_2_, and to the measured detectable penetration depth, *d* with the following equation, *r*_1_^2^ = *r*_2_^2^ + (*r*_2_ - *d*)^2^, which is derived from the geometric Pythagoras theorem (Figure 5B). The physical picture is that only a small portion of the bead is in contact with the gold sensor surface, while most of the bead resides above the surface, and above the surface plasmon generated evanescent wave. As the evanescent wave extends into the water media, the bead, which has a measurably different refractive index from water, is partially sampled by the evanescent wave. The distance at which the refractive index change due to the presence of the bead is detectable above the background is the threshold which we interpret as the detectable extent of the surface plasmon penetration depth. Figure 5C shows a plot summarizing the image analysis routine, (and which is described in further detail in the Methods), to determine the penetration depth values. Essentially, for each SPR image, the background region of the image is used to determine the standard deviation (σ) of background noise. Intensity values of 3σ from the average background intensity are considered to be signal resulting from detectable bead material in the evanescent field. The *r*_*2*_ value is obtained from the apparent area of the object in the SPR image estimated as a circle. The SPR penetration depth for each wavelength is determined using the *r*_*2*_ value obtained for each SPR image, along with the *r*_*1*_ value obtained for the bead diameter measured from the bright field image. Figure 5D shows a theoretically calculated SPR evanescent wave intensity decay, described by a single exponential decay function, as a function of distance from the surface for 620 nm excitation light (see Methods). This plot also indicates the distance from the surface where the field decays to 1/e of its original intensity or 37% field intensity, and where the field decays to 5% field intensity, which represents the theoretically maximum distance to detect a change of refractive index. Finally, Figure 5E shows a compilation plot that depicts the calculated penetration depth and detection threshold for a range of SPR excitation wavelengths, and is also plotted with the measured detectable penetration depths for several types of polymer microspheres over the same range of wavelengths. As can be seen, the measured limit of detection, within the experimental noise, seems to be described well by the theoretically determined distance at which the evanescent field decays to 1/e (63%). This occurs for a variety of polymer microspheres with differing refractive index values (n = 1.345 to 1.59). This implies that subcellular structures observed in the SPR images (Figure 3A) are likely to reside within a distance from the surface up to a maximum amount as described by the 1/e distance threshold. This is expected to be true for all the observable cellular components (membrane, fibrillar structures, etc.) since the refractive indices of these components will be within the range of refractive indices we probed with the polymeric beads.

## Discussion

### Improvements to resolution of SPR imaging

The experimental configuration for SPR imaging shown here has several advantages over previously reported methods. Most SPR imaging optical geometries are constructed in attenuated total reflection prism-coupling Kretchmann configurations [10]. While this allows for a relatively large field of view, up to the centimeter scale, the spatial resolution with this approach is inherently limited for several reasons. First, the oblique angle of the sample plane to the imaging plane skews one dimension relative to the other and needs to be corrected optically or through imaging analysis [23, 24]. Second, is the limit in magnification due to imaging through a prism.

The use of an objective lens for SPR imaging provides greater magnification and resolution compared to the prism-based system [25]. The objective-based SPR system eliminates the spatial distortion that occurs in a prism-based system by keeping the object plane parallel to the image plane. There are some compromises associated with this configuration, one of which is the relatively small field of view (generally < 100 μm) and the requirement for specialty high numerical aperture objectives and matching coverslips. In general, a special 1.65 NA objective is necessary to be able to launch incident light at angles beyond the critical angle under aqueous media [26].

We show here that we achieve a lateral resolution for SPR imaging in the axis perpendicular to the direction of surface plasmon excitation of 0.3 μm, which is approximately equivalent to the theoretical limit. However, one limitation for imaging remains and that is the erosion of resolution in the direction of propagation of the excited surface plasmon wave. We show here that high resolution can be achieved in both directions, parallel and perpendicular to the direction of plasmon propagation, by using a limited range of azimuthal angles to excite SPR. While it has been shown from both theory and measurements [25, 27] based on a discrete incident angle that resolution in the direction of SP propagation is ≈ 3 μm for 620 nm light, it has also been shown that a combination of angles can lead to shorter apparent propagation lengths perhaps due to optical interference [28]. Here we show that the resolution in the direction of the surface plasmon propagation in this system is 0.6 μm (Figure 4B), indicating the significance of this effect. This resolution allows the observation of sub-micrometer features in cellular and non-cellular structures near the cell-substrate interface.

In contrast to the typically used uneven coherent laser illumination sources, here we use the incoherent light of a digital light projector to provide even illumination and to optically control incident angle and azimuthal length. This feature of the design significantly contributes to the high level of optical resolution we have observed here.

### High numerical aperture polarization aberrations and SPR image analysis

Previous work using a prism in the Kretchmann configuration for SPR imaging has shown that dividing p- by s-polarized light normalizes reflectivity and reduces spatial noise in incident light intensity [13]. However, in the present configuration using an objective lens, there appears to be an aberration in the normalized reflectivity. This is due to diattenuation between the p- and s- components that pass through steep optical interfaces; the s-polarized light no longer remains directly proportional to the incident light at high incident angles [29]. Methods for correcting for this effect will be described in detail in a separate communication, but for the data shown here, the intensity in each SPR image is normalized to the reflectivity value for the corresponding angle on a Fresnel model fit to the SPR angle scan taken from the BFP.

### SPR penetration depth and limit of detection

SPR signal contrast can be due to mass or to refractive index changes near the interface. There have been few needs to test the limits of the surface plasmon penetration depth, but it is important for cell measurements that are made with SPR. In the SPR literature, the evanescent wave penetration depth is often derived from calculations assuming a 1/e decay length, and experimental measurements made using Raman scattering have demonstrated a decay length in agreement with theory [30, 31]. The decay length, however, does not describe a finite limit of detection. Previously, a fluorescent microsphere with a refractive index that was matched to the media was used to evaluate the shape of the exponential decay for the evanescent field for total internal reflection fluorescence microscopy (TIRFM) [32]. Here we use polymer microspheres with refractive index similar to biological media in water as cellular mimics to directly measure the limit of detection of an object with surface plasmons as limited by penetration depth of the evanescent field. With conditions similar to the cell environment, they serve as useful calibration aids for quantifying cellular material that resides at a distance above the substrate in the cell. Additionally, the measured dependence of penetration depth on the wavelength of incident light allows one to control and probe specific distances away from the substratum at a scale of tens of nanometers. These measurements appear to confirm that the 1/e detection limit for SP excitation is a reasonable estimate, and that there is a clear dependence of penetration on wavelength of excitation. This knowledge will likely be important for interpreting SPR images of cells, for example, determining the distance between the nucleus and the substratum, and suggests that many intracellular structures reside close to the interface.

Another technique that involves coupling of light energy into a surface layer, TIRFM, can excite fluorescence well beyond a distance of 1/e from the surface. While some of that signal may be due to scattering [32, 33], the apparently deeper penetration may also be due to lower background for fluorescence than for reflectivity. Sources of background measurement noise in the SPRI image include inhomogeneities in coverslip or media refractive index, and gold roughness. In our experiments, we minimized noise inherent to the imaging process by acquiring and averaging multiple, identical images. Another plausible explanation for observing a penetration depth much less than reported for TIRFM is that the model used to estimate the exponential decay of the SP signal and the 1/e distance may not be accurate. The theoretically calculated penetration depths shown here for comparison rely on the fidelity of the optical constants used for the gold layer. There is known to be some deviation of the dielectric constant for thin gold films compared to constants reported for bulk gold, and additional deviation occurs upon chemical modification of the gold [34, 35]. Therefore, there may be other unaccounted for reasons for observing the 1/e distance detection limit.

Our measurements of penetration depth were highly consistent despite differences in microsphere refractive index and size. For example, when comparing two microspheres with notably different refractive index values, Sephacryl (*n* = 1.345) and silica (*n* = 1.420), both types of beads give similar resulting penetration depth values, yet the silica beads have a larger ΔR signal intensity than Sephacryl (data not shown). The likely explanation for this observation is that the ΔR signal intensity can become non-linear and saturate at higher refractive index changes, a known phenomenon for SPR imaging. As a consequence, the potential signal-to-noise that could be gained using higher refractive index microspheres is not realized. Our penetration depth measurements were uniform across different sized beads, suggesting the absence of any systematic bias in the method due to bead size.

## Conclusions

We present a versatile, high resolution surface plasmon resonance imaging architecture that enables access to the SPR imaging plane and the back focal plane, along with access to a range of excitation wavelengths. This flexibility in imaging modes will be important when making measurements in complex cellular environments and enable optimization for exploring cellular structures and secretions. Although not demonstrated here, the technique can be applied to living cells to collect dynamic data in a rapid and noninvasive manner. The use of patterned light to achieve precise angle control with a DLP nearly eliminates the spatial aberrations that normally affect SPR imaging. The ease of scanning and selection of angle of incidence with computer control will enable automation, reproducibility, and hyperspectral SPR imaging by allowing acquisition of the complete SPR response curve at each spatially resolved pixel. Additionally, comparisons to other image modes are now facilitated due to adaption on a microscope base system and comparable diffraction limited resolutions. The unprecedented subcellular detail observed here created the need to fully understand the measurable extent of the SPR evanescent wave. By using polymer microspheres as a tool to probe the penetration depth, an effective finite detectable field limit has been determined, and will aid in quantitative interpretation of cellular and subcellular imaging. Future studies of polymer microspheres as intensity calibration materials will be pursued to provide quantitative determination of the relationship of distance-from-substrate and signal intensity.

## Methods

### SPRI apparatus

The SPR through-the-objective imaging system was assembled on an inverted microscope (Olympus IX-70, Center Valley, PA) with several modifications, Figure 1. The illumination source is a digital light projector (Dell 3300MP; Dell, Round Rock, TX) that is controlled with a laptop computer using image presentation software (Power Point, Microsoft, Redmond, WA) to project specific curved- or crescent-shaped images onto regions of the back aperture, or filling the entire back aperture of the objective with the illumination. The projector lens was removed and replaced with a 4X objective (Edmund Optics, Barrington, NJ). The focused light was collimated with an achromatic lens (*f* = 60 mm) and directed through a bandpass filter (FWHM = 10 nm; Thorlabs, Newton, NJ), and a rotatable linear polarizer (Thorlabs). This polarized monochromatic light is directed into a customized tube lens (*f* = 100 mm), through a filter cube mounted with a pellicle beam splitter (Thorlabs), and onto the back focal plane (BFP) of a high numerical aperture (NA) objective (100×, 1.65 NA, Olympus). From the focal lengths of the collimating lens and the tube lens, and the distances between them and the image plane, the magnification of the projected image onto the BFP of the objective is determined to be 1/3 according to the thin lens formula [36]. The specific location of the shaped object on the computer screen controls the corresponding position of the light shaped object on the BFP of the objective and subsequently the particular incident angle of the incident light on the gold coated coverslip (No. 0, Olympus). The coverslip and the objective are coupled with refractive index (*n*) matching fluid, *n* = 1.78 (Cargille Laboratories, Cedar Grove, NJ). The incident light couples to the plasmons in the gold film and a fraction of the p-polarized light is reflected or absorbed depending on the angle of incidence. The reflected SPR image is directed out the microscope body, through a lens assembly that can be positioned in or out of the beam and onto a 12-bit Coolsnap FX CCD camera (Roper Scientific, Tucson, AZ). The CCD camera images the back focal plane; with the lens assembly positioned in the optical path, the image plane is focused onto the CCD. The first lens of the assembly selects for the image plane, and the second lens adjusts the image magnification. Images were acquired from the CCD using Micro-Manager (https://www.micro-manager.org/) open source microscopy software.

### Substrate preparation

Coverslips (18 mm diameter, *n* = 1.78, Olympus) were acid washed with 7:3 (v/v) H_2_SO_4_:H_2_O_2_, rinsed with 18 MΩ⋅cm distilled water, rinsed with ethanol, dried, and then coated with ≈ 1 nm chromium and ≈ 45 nm gold (99.99% purity) by magnetron sputtering using an Edwards Auto 306 vacuum system (Edwards, Wilmington, MA) at 1 × 10^-7^ mbar. For microsphere based experiments, a static fluidic chamber made out of polydimethylsiloxane (PDMS) was assembled on top of the coverslip, and the bare gold surface under distilled water was used as the substrate and media. For cell based experiments, the gold coated coverslip was immersed in a 0.5 mmol/L hexadecanethiol solution in ethanol for 12 h to generate a self-assembled monolayer. The coverslip was then inserted into a sterile solution of 25 μg/mL bovine plasma fibronectin (Sigma, St. Louis, MO) in Ca^2+^- and Mg^2+^- free Dulbecco’s phosphate buffered saline (DPBS; Invitrogen, Carlbad, CA) for 1 h.

### Cell culture

The rat aortic vascular smooth muscle cell line, A10 (ATCC, Manassas, VA), and the mouse embryo fibroblast NIH 3T3 line (ATCC) were maintained in Dulbecco’s Modified Eagles Medium with 25 mM HEPES (DMEM; Mediatech, Herndon, VA) supplemented with nonessential amino acids, glutamine, penicillin (100 units/mL), streptomycin (100 μg/mL), 10% by volume fetal bovine serum (FBS ) (Invitrogen, Carlsbad, CA); the human lung carcinoma cell line A549 (ATCC) was maintained in RPMI medium (Invitrogen, Carlsbad, CA) supplemented with glutamine, penicillin (100 units/mL), streptomycin (100 μg/mL), 10% by volume FBS (Invitrogen, Carlsbad, CA); the human hepatocellular carcinoma Hep G2 and African green monkey kidney Vero lines (ATCC) were maintained in Eagle’s Minimum Essential Medium (EMEM) containing 1.0 mM sodium pyruvate, 0.1 mM non-essential aminoacids, 1.4 g/L sodium bicarbonate (ATCC) supplemented with glutamine, penicillin (100 units/mL), streptomycin (100 μg/mL) and 10% by volume FBS . All cell lines were maintained in a humidified 5% CO_2_ balanced-air atmosphere at 37°C. Cells were removed from tissue culture polystyrene flasks with 0.25% trypsin-EDTA (Invitrogen), and were seeded in culture medium onto the fibronectin coated substrates at a density of 1000 cells/cm^2^. After 72 h incubation, cells on the substrates were washed with warm Hanks Balanced Salt Solution (HBSS; ICN Biomedicals, Costa Mesa, CA), fixed in 1% (v/v) paraformaldehyde(EMS, Hatfield, PA) in DPBS for 30 min at room temperature, quenched in 0.25% (m/v) NH_4_Cl in DPBS (15 min) and rinsed with DPBS. After rinsing with DPBS, the fixed cell substrates were overlaid with a fluidic chamber made out of polydimethylsiloxane (PDMS) and kept under DPBS for all microscopy measurements.

### Polymer microspheres

Polymer microspheres of various materials and sizes were obtained from the following sources: silica microspheres (refractive index (*n*) = 1.42, diameter 6.1 μm; Bangs Laboratories, Inc., Fishers, IN), poly(methyl methacrylate) (PMMA) microspheres (*n* = 1.48, diameter 63 μm to 75 μm; Cospheric, Santa Barbara, CA), Sephacryl S-300 microspheres (*n* = 1.345 (estimate), diameter 25 μm to75 μm; GE Healthcare Biosciences, Pittsburgh, PA), polystyrene -latex microspheres (*n* = 1.59, diameter 43 μm; Beckman Coulter, Miami, FL), polystyrene-latex microspheres (*n* = 1.59, diameter 5.7 μm; Molecular Probes, Inc., Eugene, OR). In most cases, ≈100 μL of stock bead suspension was diluted 1/10 in distilled water. This dilution was centrifuged and then resuspended with 1 mL nanopure distilled water, repeated twice. A fraction of the distilled water-bead suspension was then added to a fluid chamber mounted on a gold coated substrate at room temperature. If the microspheres were shipped dry, the first step was to add a small volume, ≈50 μL, to a microfuge tube and resuspend in 1 mL distilled water. The green fluorescent microspheres (diameter (0.175 ± 0.005) μm; Life Technologies, Grand Island, NY) used to test instrument resolution were used as received in aqueous suspension.

### Phase contrast, bright field, and fluorescence microscopy

Phase contrast microscopy images were acquired using a 20X, 0.4 NA, Ph1 objective (Olympus) on the same microscope used for SPRI. The SPR image was registered to the phase contrast image using 2 fiduciary marks according to the TurboReg plugin in the ImageJ software. The epi-fluorescence images of the polymer microspheres were acquired with a 100X, 1.65 NA objective, the excitation light was generated by the DLP fully illuminated using a 480 nm excitation filter (480 nm, FWHM = 10 nm; Thorlabs). For emission collection, a FITC filter (530 nm, FWHM = 43 nm; Thorlabs) was inserted into the lens assembly before the CCD. A micrometer scale reference was imaged under bright field conditions and used to calibrate the spatial dimensions of the pixels. Bright field imaging of polymer microspheres were performed in transmission mode using the same 100X, 1.65 NA objective as for SPRI.

### Fluorescence staining and imaging

Subsequent to SPR imaging the previously fixed A10 cells were permeabilized with 0.05% TX-100 in PBS and blocked in 10% goat serum/1% BSA in PBS (blocking solution) for 30 minutes at room temperature. The sample was then stained with monoclonal anti-vinculin antibody (Sigma) diluted 1:200 in blocking solution for 1 hour at room temperature. The sample was then rinsed 3X with PBS and blocked again with 10% goat serum/1% BSA in PBS for 30 minutes at room temperature. The sample was then stained with Alexa-488 goat anti-mouse secondary antibody (Invitrogen) diluted 1:100 in blocking solution for 45 minutes at room temperature. Finally, the sample was rinsed 3× in PBS and stored in PBS for imaging. Fluorescence images were acquired with a 1.3 NA, 63X objective on an upright Zeiss microscope (Zeiss, Jena, Germany) using a standard FITC filter cube set. The fluorescence image was registered to the SPR image using 2 fiduciary marks according to the TurboReg plugin in the ImageJ software.

### SPR image collection and analysis

For SPR back focal plane (BFP) imaging, the digital light projector (DLP) was set to project non-patterned, white light illumination. The excitation wavelength was selected with a rotatable band-pass filter wheel (670 nm, 620 nm, 590 nm, 550 nm, 515 nm, 480 nm, FWHM = 10 nm; Thorlabs). The lens assembly before the camera was switched out to project the image on the BFP onto the camera CCD. A homogeneous material sample, in our case a gold coated coverslip with a water filled chamber, was mounted via coupling fluid on the objective. An image was acquired with the linear polarizer oriented in the x-image direction, which we term the p-polarized light image, as the SPR minima is visible at the periphery of the BFP in the x-axis but not in the y-axis. A line scan of the region of interest (ROI) was selected along the x-axis of the p-polarized light image from the center to the periphery of the BFP provides reflectivity values as a function of angle of incidence. In other configurations, this information would be provided by a sequential angle scan. These intensities were normalized to reflectivity units from a SPR curve fit with a 3-layer Fresnel model using literature values for the optical constants of the layer involved (glass/gold/media) [15–18].

Prior to SPR imaging of the specimen, the incident angle of light selection was optimized in the BFP. After mounting and aligning the sample, the DLP was set to fully illuminate the BFP with p-polarized light at selected wavelength as described above. The shape of the light projection was then changed to a thin crescent shaped beam of light of ≈ 40 arc degrees in the BFP circle. The crescent shape was initially placed near where the SPR minimum was visualized when fully illuminated with non-patterned light. Switching the image view from the BFP to the sample plane, using the microscope ocular or CCD camera, the crescent shape position was then finely tuned by translating the crescent shape in the x-direction in the BFP until the intensity value in the image plane was minimized. The minimum intensity value across the range of angles determined by the crescent is ≈ 0.05 reflectance. The crescent shape was then adjusted slightly shallow of the SPR minimum, to a reflectance minimum of ≈ 0.1 reflectance, to ensure that the subsequent SPR imaging response will be in the linear response range [37]. In this way, by using the BFP image, one can obtain the reflectivity response versus incident angle over a range of angles simultaneously, and from the SPR sample plane one can obtain the average reflectivity value in the image intensity. For experiments on bare gold surfaces in distilled water, the incident angle for maximum SPR coupling was calculated to be ≈ 54.0° for 620 nm. The process of incident angle selection was performed for each selected wavelength above.

For each SPR image in the sample plane a p- and s-polarized image was taken by rotating the linear polarizer 90° and using a crescent shape of light near the SPR minimum optimized as described above. Dividing the p- by s-polarized image reduces the effect of spatial inhomogeneity in incident light, a strategy adapted from a previous SPRI prism configuration [13]. The resulting image was normalized to reflectivity units based upon an SPR angle scan. For subsequent analysis and comparison, the images were further modified to convert the reflectivity units into Δ-reflectivity (ΔR) by using *Δ*R = R_1_–R_0_ where a background ROI under water or buffer media is used to calculate R_0_ which is then subtracted from the rest of the image values, R_1_, to convert to ΔR.

For determination of the measured radius of microspheres in the SPR images, a script was written in ImageJ that uses a manually selected circular ROI approximately centered on the microsphere. The ROI extends several micrometers from the detectable outside edge of the microsphere. The circle diameter was then dilated by 10 μm and all pixel values between the two circled areas were used to calculate the standard deviation (σ) of the image background. A threshold with a value of 3σ was used to delineate background; the number of pixels above the threshold was used to compute the area and radius of the microsphere object within that area.

The evanescent wave decay profile was calculated by a single exponential decay function I(z) = I_0_e^(-z/Lp)^ where z is the distance perpendicular from the surface and the initial intensity I_0_ = 1. The penetration depth at 1/e (L_p_) is calculated according to the formula for surface plasmon penetration depth [21] with published optical properties for gold and water [16, 38] for each wavelength measured here (670 nm, 620 nm, 590 nm, 550 nm, 515 nm, 480 nm). All image analysis was performed using ImageJ software (http://rsb.info.nih.gov/ij/). Angle dependent SPR data were analyzed using stock and custom code written in MATLAB (Mathworks, Natick, MA).

## Electronic supplementary material

Additional file 1: **Demagnification and projection of the incident light image onto the back focal plane.** (PDF 432 KB)

Additional file 2: **SPR angle scan obtained from BFP compared with optical model.** (PDF 176 KB)

Additional file 3: **Optimization of crescent shaped DLP pattern of light in BFP for SPR imaging.** (PDF 502 KB)
