# Supplementary material for: High resolution surface plasmon resonance imaging for single cells
Source: BMC Cell Biol. 2014 Dec 1;15:35. doi: 10.1186/1471-2121-15-35 (PMC4289309; doi:10.1186/1471-2121-15-35)
Supplement: Supplementary file 1 — Additional file 1: Demagnification and projection of the incident light image onto the back focal plane. (PDF 432 KB) [file 12860_2014_726_MOESM1_ESM.pdf]

Additional file 1:

Demagnification and projection of the incident light image onto the back focal plane

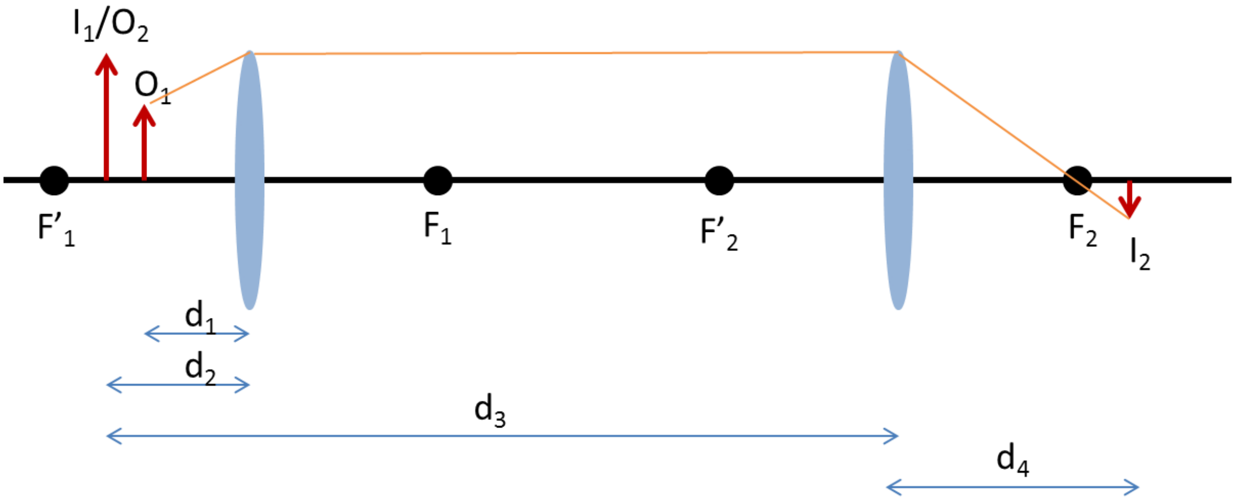

F_1 = focal length of collimating lens
F_2 = focal length of short focal length lens
d_1 = distance to object
d_2 = distance to (virtual) image created by first lens
d_3 = distance to object (virtual image 1) for second lens
d_4 = distance to image created by second lens
M_1 = lens magnification from first lens
M_2 = lens magnification from second lens
O_1 = height of object 1, the image projected from the DLP (object 1 is within the focal distance of the collimating lens)
O_2 (I_1) = height of virtual image 1, which serves as object 2 for the short focal length lens
I_2 = height of the image projected from the DLP after being demagnified by the short focal length lens.

The 'object' in this calculation is the image projected out of the DLP projector through a 4X objective (which is not shown). The 'image', I_2, is the conjugate plane of the DLP projected image; light passes through focal point, F_2 and the image is projected in focus onto the back focal plane (as indicated), which is the back surface of the 1.65 NA objective lens that focuses the incident light onto the sample plane.

The thin lens formula relates the distances of the object and image to the lens with the lens focal length:

$$\frac{1}{F_1} = \frac{1}{d_1} + \frac{1}{d_2}$$

$$\frac{1}{6\text{ cm}} = \frac{1}{2\text{ cm}} + \frac{1}{d_2}$$

d_2 = -3 cm

For the first lens, the image is located – 3 cm behind the first lens creating a virtual image as the object is closer to the lens than the focal length.

$$\frac{1}{F_2} = \frac{1}{d_3} + \frac{1}{d_4}$$

$$\frac{1}{10\text{ cm}} = \frac{1}{55\text{ cm}} + \frac{1}{d_4}$$

d_4 = 12.2 cm

For the second lens, the image is projected 12.2 cm from this lens.

$$M_1 = -\frac{d_2}{d_1}$$

$$M_1 = -\frac{(-3\text{ cm})}{2\text{ cm}}$$

M_1 = 1.5

The magnification from the first lens is 1.5.

$$M_2 = -\frac{d_4}{d_3}$$

$$M_2 = -\frac{12.2\text{ cm}}{55\text{ cm}}$$

M_2 = - 0.22

The magnification of the second lens is 0.22, and the negative value indicates that the image is inverted.

From the following relations and substitution:

$I_1 = O_2$, $I_1 = M_1 \cdot O_1$, $I_2 = M_2 \cdot O_2$

$M_1 \cdot O_1 = I_2 / M_2$

We get the total system magnification:

$M_1 \cdot M_2 \cdot O_1 = I_2$

$(1.5) \cdot (-0.22) \cdot O_1 = I_2$

$- 0.33\ O_1 = I_2$

The total magnification of the image is – (0.33X), a negative number, indicating an inverted and smaller image than the original object.
