# Supplementary material for: High resolution surface plasmon resonance imaging for single cells
Source: BMC Cell Biol. 2014 Dec 1;15:35. doi: 10.1186/1471-2121-15-35 (PMC4289309; doi:10.1186/1471-2121-15-35)
Supplement: Supplementary file 2 — Additional file 2: SPR angle scan obtained from BFP compared with optical model. (PDF 176 KB) [file 12860_2014_726_MOESM2_ESM.pdf]

Additional File 2:

**SPR angle scan obtained from BFP compared with optical model**

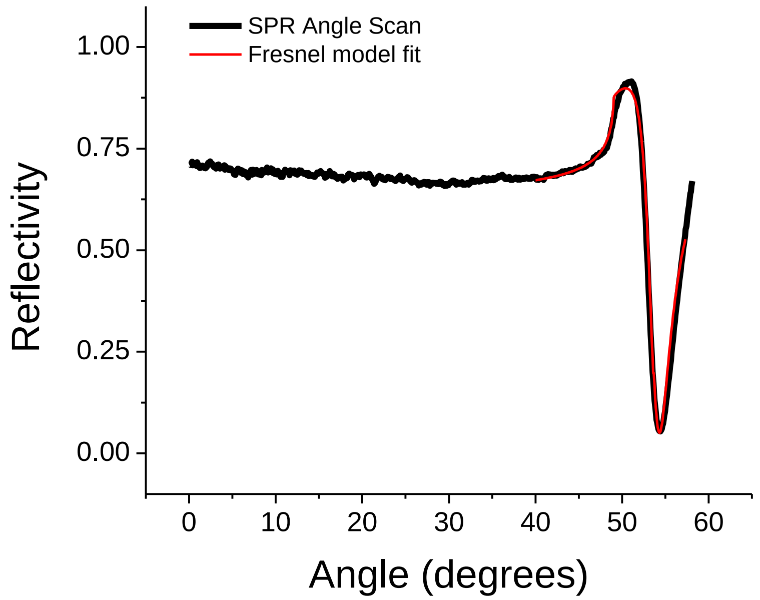


A line scan across the center of the BFP to the periphery (0° to 60°) in the p-polarized direction displays the SPR dependent angle scan. Overlaid on the SPR data is a fit from the SPR curve generated by the Fresnel optical model using the literature values for a prism/gold/water interface.
