# Supplementary material for: High resolution surface plasmon resonance imaging for single cells
Source: BMC Cell Biol. 2014 Dec 1;15:35. doi: 10.1186/1471-2121-15-35 (PMC4289309; doi:10.1186/1471-2121-15-35)
Supplement: Supplementary file 3 — Additional file 3: Optimization of crescent shaped DLP pattern of light in BFP for SPR imaging. (PDF 502 KB) [file 12860_2014_726_MOESM3_ESM.pdf]

Additional File 3:

Optimization of crescent shaped DLP pattern of light in BFP for SPR imaging

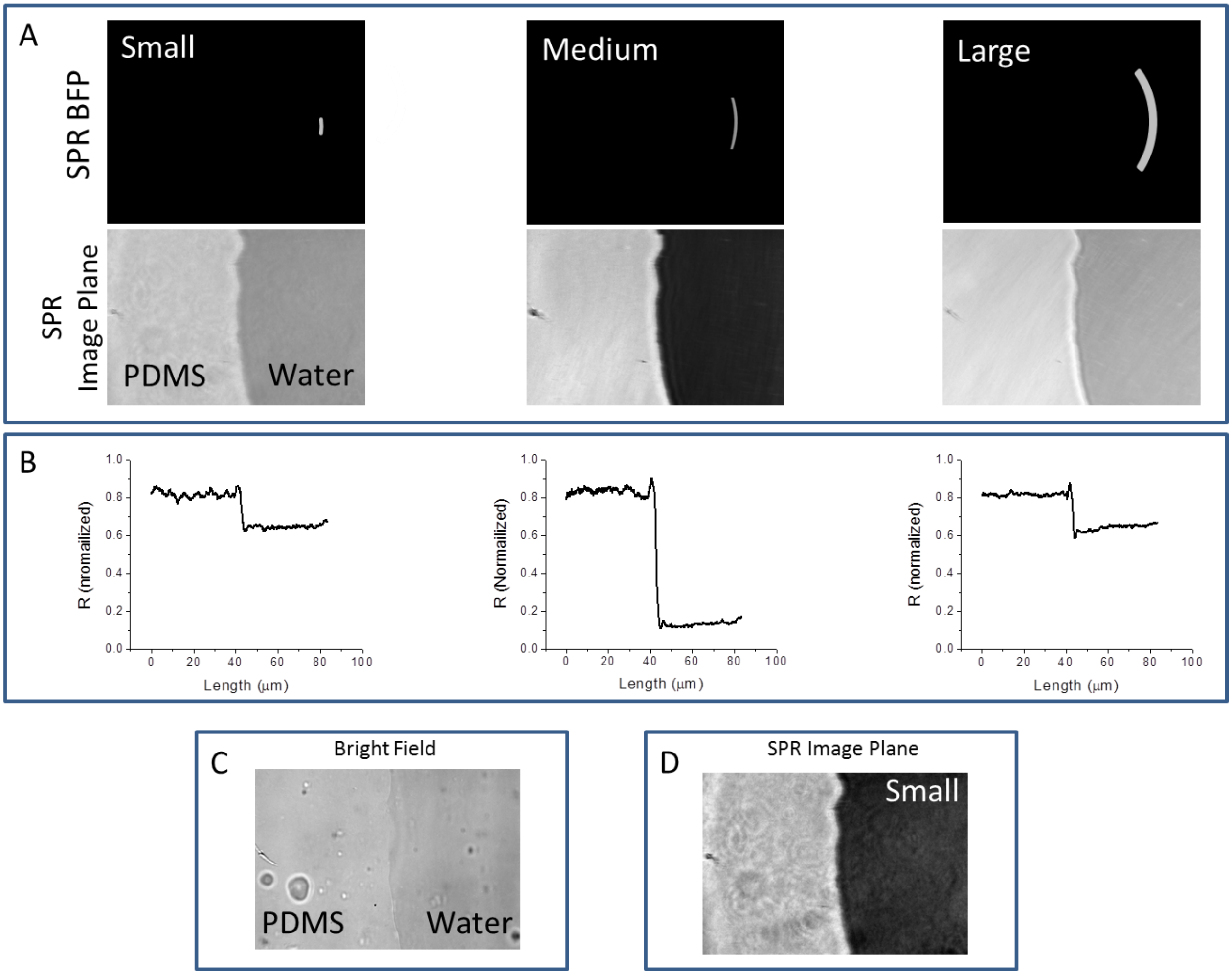

A. BFP images are shown for 3 sizes of crescent shapes used for SPR excitation and their corresponding SPR generated images for a sample of polydimethylsiloxane (PDMS) adjacent to water on a gold coated coverslip. The medium-labelled shape is ≈40° arc length, and is positioned ≈53.5° incident to sample. The resulting SPR images show the level of contrast between PDMS and water on a normalized reflectivity scale from 1 to 0.
B. A line scan is taken horizontally across each SPR image and is displayed as reflectivity (R) intensity normalized to the reflectivity of the medium-labelled sample image from 1 to 0. The medium-sized shape appears to have the best image quality and contrast. In comparison to the medium-sized shape, the large-sized shape results in an SPR image with decreased contrast presumably due to increased background from illuminating light that does not contribute to the SPR signal. The small-sized shape also produces an image with reduced contrast. This is likely due to an increase in background noise resulting from a longer required exposure time.

Additionally, the image quality looks to be further reduced by the appearance of interference patterns.

C. Comparison image of sample (PDMS/water) illuminated under bright field.
D. Contrast adjusted SPR image resulting from the small-labelled projected shape to better show the interference artifacts.
